# Emerging Implantable Sensor Technologies at the Intersection of Engineering and Brain Science

**DOI:** 10.3390/bios15110762

**Published:** 2025-11-17

**Authors:** Lihong Qi, Yuheng Wang, Xuemei Liang

**Affiliations:** 1 Department of Geriatrics, School of Clinical Medicine, Southwest Medical University, Luzhou 646000, China; 2 The Faculty of Electrical Engineering and Computer Science, Ningbo University, Ningbo 315211, China; 3 Functional and Molecular Imaging Key Lab of Shaanxi Province, Department of Radiology, Tangdu Hospital, Air Force Medical University, Xi’an 710032, China; 4 Neuromodulation Center, Chongqing Brain and Intelligence Science Center, Chongqing 401331, China

**Keywords:** flexible neural interfaces, wireless & bioresorbable systems, multimodal sensing, brain–machine interface, neuromorphic electronics

## Abstract

Advances in implantable sensor technologies are revolutionizing the landscape of brain science by enabling chronic, precise, and multimodal interfacing with neural tissues. With the convergence of material science, electronics, and neurobiology, flexible, wireless, bioresorbable, and multimodal sensors are expanding the frontiers of diagnosis, therapy, and brain-machine interfacing. This review presents the latest breakthroughs in implantable neural sensor technologies, emphasizing bio-integration, signal fidelity, and functional adaptability. We highlight innovations such as CMOS-integrated flexible probes, internal ion-gated organic electrochemical transistors (IGTs), multimodal neurotransmitter-electrophysiology sensors, and wireless energy systems. Finally, we discuss the clinical potential, translational challenges, and future directions for brain science and neuroengineering. We further benchmark transduction and analytical performance in physiological media and outline in vivo calibration, antifouling/packaging, and on-node data-efficient processing for long-term stability.

## 1. Introduction

Brain science increasingly relies on implantable sensor technologies to decode neural activity, modulate neural circuits, and restore lost physiological functions [1,2,3,4,5,6,7,8,9,10,11,12,13,14,15,16,17,18,19,20,21,22,23,24,25,26,27,28,29,30]. From epilepsy monitoring and Parkinson’s symptom control to neuroprosthetic feedback and spinal cord injury rehabilitation, implantable devices are emerging as critical tools that bridge the gap between neurobiology and clinical neurology. Recent developments have rapidly advanced flexible and high-density electrode arrays [31,32,33,34,35,36,37,38], bioadhesive and regenerative materials [39,40,41,42,43,44,45,46,47], conductive polymer and stability engineering [48,49,50,51,52,53,54,55], and therapeutic bioelectronics for wound repair and neural modulation [56,57,58,59,60,61,62,63,64,65,66,67], while also addressing ethical, AI, and fabrication considerations [68,69,70]. This review is complemented by recent advances in wearable and motion-based sensing technologies, which offer important insights for both activity monitoring and neurodegenerative disease assessment [71,72].

The evolution of neural interfaces has paralleled the broader miniaturization and functional integration trends in electronics and materials science. Historically, rigid metal and silicon electrodes were used to record neuronal signals, but these often led to mechanical mismatch with soft neural tissues, triggering immune responses and signal degradation. In recent years, the convergence of soft materials, ultrathin electronics, and bioresorbable components has opened up transformative possibilities for long-term brain-machine interfacing. Additionally, the integration of wireless communication, closed-loop stimulation, and AI-assisted decoding has begun to shift these technologies from laboratory settings to potential clinical reality.

These technological advances are especially timely given the growing burden of neurological disorders. Alzheimer’s disease, Parkinson’s disease, epilepsy, stroke, and traumatic spinal cord injuries collectively affect hundreds of millions of individuals worldwide. Their incidence is rising with aging populations and increased longevity. Current diagnostic tools are often invasive, episodic, or insensitive to early pathological changes. Therapies are often generalized and reactive rather than personalized and adaptive. This creates a critical demand for innovative solutions that enable earlier diagnosis, long-term monitoring, and responsive neuromodulation tailored to individual neurophysiological patterns.

Implantable neural sensors address this need by offering continuous, in vivo access to both electrical and biochemical signatures of brain activity. Unlike surface EEG or imaging modalities, they provide cell- and circuit-level insights into dynamic brain states. Importantly, next-generation sensor technologies are not merely miniaturized—they are designed to be bio-integrative, functionally adaptive, and compatible with the complex tissue microenvironment of the brain.

To facilitate translational progress and comparative assessment across diverse sensor platforms, we propose a unifying framework that captures key engineering and regulatory considerations. This includes bio-integration, front-end signal fidelity, energy management, and data efficiency—critical dimensions that directly influence clinical readiness. Representative platforms such as CMOS-integrated flexible probes, IGT/OECT arrays, wireless and bioresorbable systems, and multimodal neurochemical-electrical sensors are examined not only in terms of device performance, but also manufacturability, safety, and long-term operational stability. Detailed performance benchmarks and translational constraints are further discussed in Supplementary Table S1, serving as a roadmap for future clinical translation.

In this review, we present a comprehensive survey of recent innovations in implantable neural sensor technologies—covering advances in soft and bioresorbable materials [1,2,3,4,5,6,7,8], neural interface and front-end circuits [9,10,11,12,13,14,15], wireless and closed-loop control systems [16,17,18,19,20], and multimodal sensing and intelligent diagnostics [21,22,23,24,25,26]. We focus on flexible electronics, wireless and battery-free systems, bioresorbable implants, and multimodal sensors capable of detecting both electrophysiological and neurochemical activity. Each category is evaluated not only in terms of technical performance but also in terms of biocompatibility, signal fidelity, and translational readiness. Our aim is to provide a unified perspective on how cross-disciplinary advances are shaping the future of brain-interface systems—enabling new avenues for diagnosis, therapy, and human–machine integration [1,2,3,6,7,8,9,17].

To provide a structured overview of the field, Table 1 summarizes five key technology categories in implantable neural sensing. For each category, representative platforms or methods are listed alongside their core advantages, including conformal biointegration, wireless functionality, neurochemical selectivity, motor control via BMI, and on-node adaptive processing. This categorization sets the foundation for the more detailed discussions that follow in this review.

## 2. Material and Device Innovations in Flexible Neural Interfaces for Chronic Biosensing

Traditional neural interfaces based on rigid silicon or metal electrodes present limitations such as mechanical mismatch with soft tissue, inflammation, and restricted spatial resolution. In contrast, emerging flexible electronics employ ultrathin, soft materials such as polyimide, parylene, and silk-inspired polymers to conform to brain surfaces and maintain stable long-term contact.

CMOS-based flexible neural probes have achieved scalable, high-density recording interfaces by integrating active amplification and multiplexing directly at the site of contact [37,38]. For instance, scalable CMOS-fabricated platforms with hundreds of microelectrodes enable simultaneous recording from distributed brain regions while maintaining a soft interface. These platforms minimize wire counts and enable integration with external processing systems, which is vital for reducing surgical complexity and ensuring data reliability [1]. The material composition of flexible neural interfaces plays a critical role in achieving long-term biointegration and signal stability [73,74]. Substrate materials such as polyimide, parylene-C, and SU-8 provide mechanical flexibility and chemical stability, making them widely adopted in microfabricated probes [73,74,75,76]. For encapsulation and insulation, parylene-C is particularly valued for its low water permeability and FDA approval history [77,78]. On the sensing layer, conductive polymers such as PEDOT:PSS and PPy (polypyrrole) offer high charge injection capacity, low impedance, and enhanced electrochemical transduction at the biotic–abiotic interface [79,80,81,82]. Their ability to interface with both ionic and electronic domains makes them suitable for amplifying neural and neurochemical signals.

Additionally, silk fibroin and other biodegradable materials have been explored as transient substrates that naturally dissolve after functional timescales, reducing the need for surgical retrieval. In contrast, inorganic semiconductors like silicon and GaN, while providing excellent electronic performance, require microstructuring (e.g., “island–bridge” architectures) to accommodate the mechanical mismatch with brain tissue. Emerging hybrid approaches combine ultrathin inorganic films with soft carriers to balance electrical fidelity and tissue compliance.

Organic electrochemical transistors (OECTs) further enhance signal fidelity by offering intrinsic amplification through ion-electron coupling in the channel. A significant milestone has been the development of complementary internal ion-gated transistors (cIGTs) with spatially controlled doping, enabling CMOS-like behavior using a single soft material. These cIGTs achieve over 200-fold amplification with MHz bandwidth and long-term in vivo stability [10,22], making them suitable for implantation in developing animals and small brains. Compared to traditional CMOS-based flexible probes, organic electrochemical transistors (OECTs), especially cIGTs, offer superior mechanical compliance and intrinsic amplification. However, CMOS technologies still hold advantages in scalability, integration density, and long-term electronic stability. In terms of technology readiness, CMOS-based neural probes are approaching TRL 6–7, with preclinical animal validation, while organic transistor-based platforms remain at TRL 3–4, requiring more robustness and material optimization for chronic use. This development represents a convergence of soft electronics and neuromorphic design, bringing the functionality of classical electronics into flexible, brain-compatible substrates.

**Figure 1:**
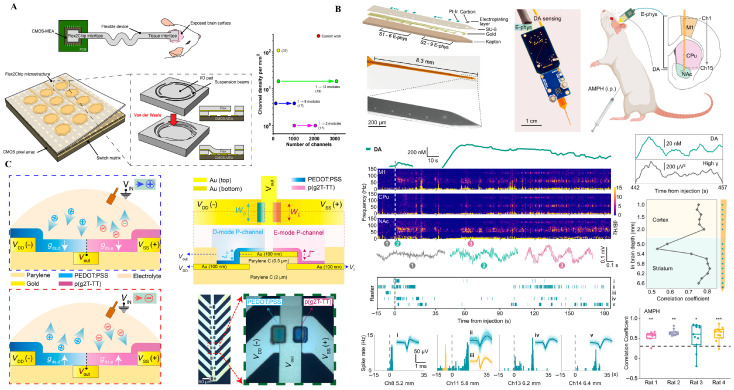
Flexible neural interface technologies. (**A**) CMOS-integrated flexible neural probe with high-density microelectrodes for distributed cortical recording. Reprinted with permission [1]. (**B**) Ion-gated organic electrochemical transistors (IGTs) using spatial doping for conformable amplification. Reprinted with permission [10]. (**C**) Neuromorphic cIGT-based amplifier array mimicking CMOS behavior for bioelectronics. Reprinted with permission [23].

Recent progress in neural interface design is exemplified by the representative systems shown in Figure 1, which highlights three distinct technological strategies—each illustrating a unique balance between integration, flexibility, and functional complexity.

Figure 1A showcases a CMOS-integrated flexible neural probe that combines large-scale microelectrode arrays with embedded signal amplification and multiplexing. This approach leverages established semiconductor technology to achieve high spatial resolution and low-noise signal acquisition. By embedding front-end electronics directly onto a soft substrate, this architecture minimizes wiring complexity and mechanical mismatch with neural tissue. However, challenges remain in thermal dissipation, long-term encapsulation, and the mechanical brittleness introduced by silicon islands, which can compromise chronic biocompatibility [23].

In contrast, Figure 1B illustrates a fully soft, organic electrochemical transistor (OECT) array utilizing ion-gated amplification. These devices, constructed from stretchable polymer semiconductors (e.g., PEDOT:PSS), operate at low voltages and exhibit intrinsic mechanical compliance with neural tissue. Their ability to directly transduce ionic activity into amplified electronic signals at the sensing interface offers substantial advantages for bio-integration. Nonetheless, limitations include relatively low carrier mobility, modest switching speeds, and environmental instability under physiological conditions [39,40,41]. Moreover, the lack of standardized fabrication processes and circuit architectures limits the scalability of OECT-based interfaces.

Pushing further toward bioinspired computing, Figure 1C presents a neuromorphic amplifier array based on complementary ion-gated transistors (cIGTs). This architecture mimics CMOS logic behavior using entirely organic materials through spatial doping control, enabling basic logic functions (e.g., inverters, NOR gates) on soft, biocompatible substrates. Such platforms promise real-time, edge-level signal processing near the biological interface, potentially enabling pre-processing, compression, or spike sorting before wireless transmission. However, this paradigm remains at a conceptual stage (TRL 2–3), with critical challenges in stability, reproducibility, and full-system integration [10].

Taken together, these approaches reflect a gradient of trade-offs between maturity and ambition [42,43,44]: from high-density silicon-based integration (Figure 1A), to soft bioelectronics with integrated amplification (Figure 1B), to fully organic neuromorphic logic (Figure 1C). Each trajectory offers unique opportunities and limitations in achieving long-term, multimodal, and intelligent brain interfaces. For instance, OECT-based flexible ECoG arrays demonstrate >200 × intrinsic amplification at <1 mW per channel, achieving sub −100 µV noise floors under chronic conditions (see Supplementary Table S1). These quantitative gains underscore the fidelity–encapsulation trade-off central to flexible bioelectronic systems. Flexible neural interfaces excel in bio-integration (B) through soft polymer substrates and conformal architectures that minimize tissue response. Their fidelity (F) benefits from CMOS and OECT amplification yet remains limited by encapsulation durability. Energy (E) efficiency is moderate, as most designs rely on wired or semi-passive configurations. Long-term drift (D) from biofouling and micromotion underscores the need for adaptive calibration and surface stabilization strategies.

## 3. Wireless and Bioresorbable Neural Interfaces for Transient and Closed-Loop Sensing

Recent progress in implantable biosensors has enabled wireless, battery-free, and bioresorbable platforms capable of monitoring electrical and physiological signals in a minimally invasive and transient manner. These systems eliminate the need for rigid leads, bulky batteries, or secondary surgeries, thereby improving patient safety, compliance, and long-term usability. Such platforms typically exploit energy harvesting through inductive coupling or NFC, backscatter communication, and biodegradable materials such as magnesium, molybdenum, silk fibroin, and PLGA polymers, ensuring both functional performance and controlled resorption after therapeutic use.

Wireless neuroprosthetic systems, exemplified by brain–spine interfaces, have shown the ability to decode cortical activity and stimulate spinal circuits, restoring volitional movement in preclinical and early clinical studies. Paired with AI-based decoders, these systems form closed-loop neuromodulatory networks capable of adapting stimulation patterns in real time. Beyond locomotor recovery, bioresorbable implants are also being investigated for post-surgical pain management, peripheral nerve rehabilitation, and inflammatory modulation, while skin-interfaced NFC devices extend applications to wound healing and rehabilitation monitoring.

At the device level, passive backscatter-based Mg sensors can report water ingress across encapsulation films with minimal energy budgets, enabling long-term diagnostic monitoring. In contrast, active bioresorbable stimulators integrate transient metals and polymers to deliver short-term neuromodulation, dissolving after function to avoid secondary extraction. This distinction highlights the trade-off between energy efficiency and functional richness: passive systems offer excellent safety margins but limited data content, while active platforms are versatile but constrained by miniaturization and power delivery.

Moreover, on-demand bioresorbable neurostimulators represent a major advance in transient electronics. These devices dissolve after serving their therapeutic function, eliminating the need for surgical removal and mitigating long-term risks. Such systems often integrate biodegradable metals, polymer substrates, and encapsulations that degrade safely in vivo. Their applications range from post-surgical nerve stimulation to temporary modulation of inflammation, offering a paradigm shift in how implantable devices are conceptualized. A critical distinction exists between passive backscatter-based systems and active wireless neurostimulators [49,50]. While passive systems exhibit excellent energy efficiency and minimal thermal load, they are limited in bandwidth and data richness. Active systems, although more versatile, pose challenges in miniaturization and energy harvesting. Clinically, wireless spinal interfaces (e.g., for locomotion restoration) are already in TRL 7–8, whereas fully biodegradable systems remain at TRL 3–4. Over the next 5 years, hybrid systems combining passive sensing with on-demand active stimulation are likely to see first-in-human trials.

**Figure 2:**
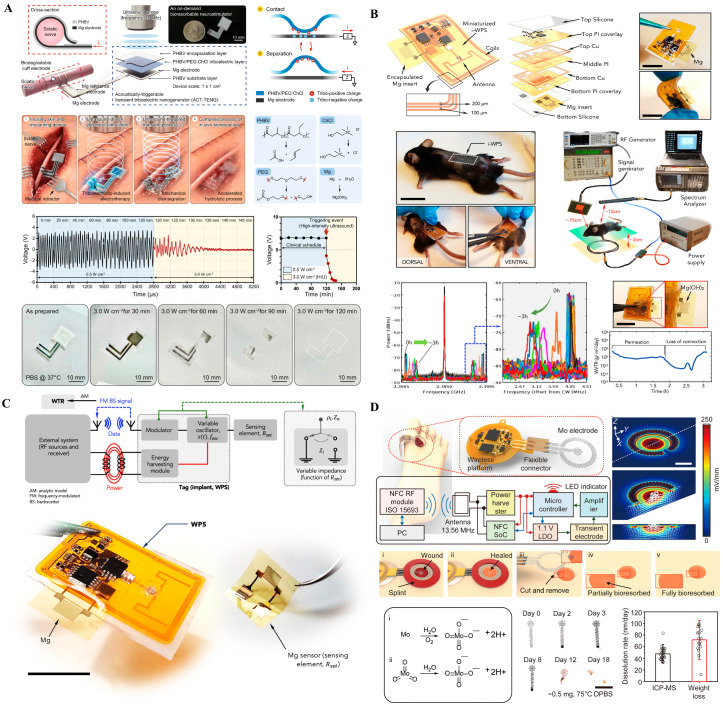
Wireless and bioresorbable implantable systems. (**A**) Magnesium-based passive backscatter sensor indicating biofluid infiltration for real-time integrity monitoring. Reprinted with permission [8]. (**B**) Bioresorbable neurostimulator designed to dissolve after temporary therapeutic use. Reprinted with permission [6]. (**C**) Wireless, battery-free wound-healing system enabling electrotherapy and impedance monitoring. Reprinted with permission [7]. (**D**) Flexible, battery-free stimulation platform using molybdenum electrodes with NFC control, demonstrating partial/complete bioresorption and programmable stimulation in a rodent model.

Wireless implants offer reduced risk of infection and greater patient comfort, while bioresorbable systems eliminate the need for secondary extraction surgeries [45,46,47,48]. Representative systems that embody these trends are illustrated in Figure 2 and are discussed below.

Figure 2A presents a passive, wireless backscatter sensor fabricated from magnesium, designed to monitor the structural integrity of implanted systems by detecting corrosion through changes in RF reflectance. This approach eliminates the need for batteries or active circuitry, enabling ultra-low-power diagnostic monitoring of encapsulation failure. However, its passive nature and single-function capacity limit its broader therapeutic utility. Additionally, precise RF readout in vivo remains sensitive to orientation and tissue interference, posing challenges for clinical deployment. Figure 2B shows a fully bioresorbable neurostimulator constructed from materials that safely degrade in physiological environments. Designed for temporary post-operative neuromodulation, such devices remove the need for secondary extraction surgery, significantly improving patient safety and compliance. While this paradigm offers substantial clinical value for short-term interventions, its fixed stimulation protocols and lack of closed-loop feedback limit its adaptability. Furthermore, ensuring consistent degradation kinetics and maintaining functional reliability over a short therapeutic window remain active areas of research [6,14]. Figure 2C depicts a skin-interfaced, wireless electrotherapy system that integrates impedance sensing with programmable stimulation for wound healing applications. Although not fully implantable, it reflects the increasing emphasis on soft, battery-free, and closed-loop systems capable of physiological feedback. Its NFC-powered operation allows fully untethered use and could be adapted to neural applications requiring superficial or minimally invasive modulation [7,8]. Yet, such systems are currently confined to relatively shallow targets and depend on precise alignment for efficient energy harvesting [7,8]. Figure 2D illustrates a flexible, battery-free neuromodulation platform utilizing molybdenum (Mo)-based electrodes for localized electrical stimulation and gradual bioresorption. The system integrates wireless control circuitry powered via 13.56 MHz NFC communication, enabling closed-loop operation without physical connections or bulky components. The platform comprises essential electronic modules, including a power harvester, microcontroller, and flexible interconnects embedded within a soft substrate, which conforms to wound or tissue surfaces.

Together, these four technologies reflect the expanding design space of implantable neural interfaces: from passive diagnostics (Figure 2A), to transient neurotherapy (Figure 2B), to closed-loop surface modulation (Figure 2C), and finally to flexible, bioresorbable stimulation platforms with integrated control (Figure 2D). They embody a spectrum of functionality, maturity, and biointegration, with a shared emphasis on minimal invasiveness, wireless operation, and adaptive material design—key priorities in the future of chronic and transient neurointerfaces. Recent wireless neurostimulators achieve power transfer efficiencies of 45–60% and operational lifetimes of 7–21 days in vivo, defining the current limit between transient operation and stable signal fidelity (see Supplementary Table S2). Such figures highlight the energy–biointegration trade-off inherent in biodegradable platforms. Wireless and bioresorbable systems emphasize energy autonomy (E) through inductive and backscatter links while maintaining strong bio-integration (B) via biodegradable and conformal materials. Fidelity (F) is inherently limited by transient operation and signal decay during resorption. Managing drift (D) through predictable degradation kinetics and automated recalibration remains key for reliable clinical translation. Together, these three technologies reflect the expanding design space of implantable neural interfaces: from passive diagnostics (Figure 2A), to transient therapy (Figure 2B), to closed-loop stimulation (Figure 2C). They embody a spectrum of functionality and maturity, with a shared emphasis on minimal invasiveness, patient comfort, and material biointegration [11].

## 4. Multimodal and Chemically Sensitive Neural Sensors

Neural computation operates not only via electrical signaling, but also through neurochemical dynamics such as dopamine or serotonin transmission. To access this layer, multimodal sensors combine electrochemical detection with electrophysiology, enabling time-aligned measurements of transmitter fluctuations and neuronal activity.

Carbon-based microelectrodes stabilized by conformal coatings enable fast-scan cyclic voltammetry (FSCV) or amperometry to track sub-second dopamine transients while simultaneously recording LFP/spiking, permitting event-resolved correlations between chemical release and network oscillations [24,51,52,53]. Typical trade-offs arise between temporal resolution (FSCV, high bandwidth) and spatial multiplexing (microarray density and addressing). Selectivity remains a core challenge due to catecholamine co-oxidation, pH shifts, and electroactive interferents; strategies include waveform encoding/chemometric deconvolution, enzyme/aptamer or permselective coatings, and nanostructured carbon to improve signal-to-fouling ratios.

Emerging neuromorphic and 2D-material devices (e.g., MoS_2_ charge-trap elements) blur the sensor–processor boundary: they exhibit plasticity-like transfer functions and can perform on-node event detection (e.g., seizure patterns) during acquisition [54,55,56]. When co-located with electrochemical/electrical front-ends, such elements support edge-level feature extraction, compression, and drift compensation, reducing the wireless data burden and enabling chemistry-informed closed-loop control. At present, multimodal probes are ~TRL 3–5 with promising demonstrations in reward circuitry and psychiatric models; short-term, diagnostic-oriented dual-mode probes are plausible candidates for near-term translation (≤5 years), provided that selectivity, stability, and in situ calibration are addressed.

Multifunctional and chemically sensitive neural sensors can be deployed as flexible arrays across cortical and subcortical targets or along gut–brain pathways, enabling time-aligned measurement of neurotransmitters and electrophysiology for systems-level neuromodulation. Coupling with microfluidics (localized sampling), optical/optogenetic stimulation (causal perturbation), and on-node preprocessing extends both what can be measured and how signals can drive latency-bounded, closed-loop control.

**Figure 3:**
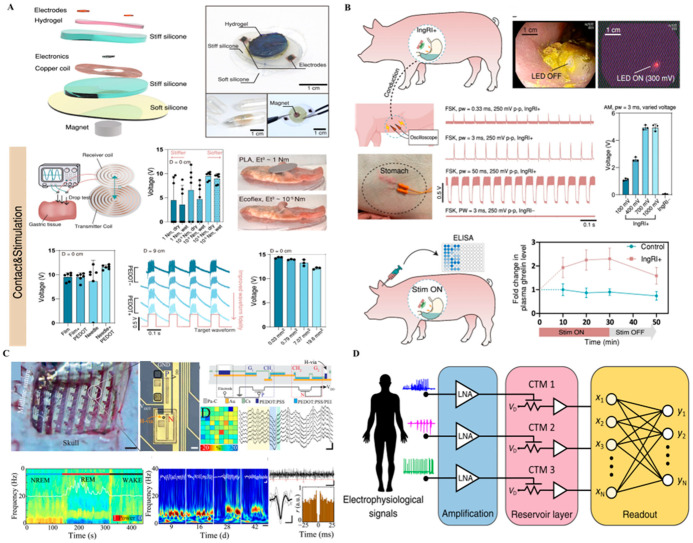
Multimodal and neuromorphic sensing platforms. (**A**) Soft ionic energy unit that provides ultra-low-power support for conformal chemical–electrical probes—appropriate for pre-amps/passive sensing where energy density demands are modest. Reprinted with permission [2]. (**B**) Ingestible, battery-free robotic interface for targeted gut–brain neuromodulation—an access route that can host localized chemical sensing and stimulation without implanted batteries. Adapted with permission [5]. (**C**) MoS_2_-based neuromorphic memory (CTM) performing edge-level pattern detection (e.g., seizures); when co-integrated with electrochemical/electrical inputs, it enables on-node classification with reduced telemetry. Reprinted with permission [22]. (**D**) Reservoir-computing architecture in which electrophysiological/chemical inputs are amplified and projected into an array of MoS_2_ CTM elements, enabling on-node feature extraction, compression, and pattern classification suitable for chemistry-informed closed-loop control.

Figure 3 highlights representative platforms that move interfaces from passive collectors toward chemistry-aware, compute-enabled systems at the bioelectronic boundary.

Figure 3A features a fully organic, ionically driven energy unit designed to support low-power soft implants. Unlike conventional batteries, this device leverages ionic gradients to generate a stable electrical output, aligning better with the aqueous, ion-rich environment of biological tissues. Its fully flexible and biocompatible form factor allows direct integration with conformal neural probes. However, the low energy density and limited scalability restrict its use to ultra-low-power applications, such as signal pre-amplification or passive sensing. Nonetheless, it represents a foundational building block for future energy-autonomous systems [2].

Figure 3B presents an ingestible, battery-free robotic platform capable of delivering targeted neuromodulation within the gastrointestinal tract. Upon activation by physiological cues, the device unfolds to establish contact with the gut lining, providing localized stimulation to enteric nerves or vagal branches. This approach enables minimally invasive access to the gut–brain axis, an emerging frontier in neural modulation. The absence of batteries and reliance on wireless magnetic control significantly reduce safety concerns. However, its applicability is currently confined to the GI tract, and challenges remain in achieving precise targeting, controlled retention time, and scalable manufacturing [5].

Figure 3C demonstrates a neuromorphic sensing element based on MoS_2_ charge-trap memory, capable of mimicking synaptic plasticity and learning behavior. When integrated with neural recording electrodes, such devices can directly perform on-site signal classification, such as real-time seizure detection. This represents a significant leap toward distributed, edge-level computation in brain–machine interfaces. Yet, stability under physiological conditions and large-scale array uniformity remain limiting factors. Moreover, system-level integration with real-time data pipelines is still in its infancy [22].

Figure 3D illustrates a reservoir-computing architecture that receives electrophysiological and electrochemical inputs, performs low-noise amplification, and projects the signals into an array of MoS_2_ charge-trap memory (CTM) elements for on-node feature extraction, compression, and pattern classification. By exploiting the history-dependent transfer characteristics of CTMs, the platform can capture transient neural or neurochemical events and deliver edge-level decisions suitable for chemistry-informed closed-loop control. This approach reduces wireless bandwidth and thermal load while increasing robustness to channel noise and motion artifacts. Remaining challenges include device-to-device variability, long-term drift and retention tunability, physiological stability of the memory states, and seamless integration with real-time data pipelines for multi-site arrays.

Collectively, the technologies in Figure 3 illustrate a shift from simple, reactive implants to devices capable of local intelligence, chemical–electrical integration, and even autonomous behavior. These developments are particularly relevant as interfaces move toward chronic, closed-loop, and context-aware operation in both central and peripheral neural systems. Quantitatively, hybrid electrochemical–electrical sensors have achieved limits of detection below 10 µM for dopamine and response times under 1 s, demonstrating high signal fidelity but also revealing baseline drift rates exceeding 2–5% per hour without in situ recalibration (Supplementary Table S2). Multimodal sensors deliver high fidelity (F) through hybrid electrochemical–electrical readout but require frequent recalibration to counter drift (D) in biochemical baselines. Enhanced bio-integration (B) is achieved using soft carbon and polymeric interfaces, while energy (E) constraints motivate neuromorphic and ionic signal processing for low-power operation.

## 5. Human–Machine Interfaces and Translational Systems

Cutting-edge implantable sensors increasingly bridge the gap between human intent and machine actuation. Brain–machine interfaces (BMIs) employing wireless cortical implants and real-time decoding systems enable volitional control of prosthetic limbs and exoskeletons. These systems have moved from basic research prototypes to early-stage clinical trials, showing promise in restoring functional independence to patients with limb loss or severe motor impairments.

In clinical contexts, wireless brain–spine interfaces have restored natural walking in spinal cord injury patients [57,58], with sensor systems decoding ECoG signals and modulating spinal stimulation in closed-loop [25]. This achievement underscores the importance of bidirectional communication between the brain and periphery in restoring volitional motor control.

Human-in-the-loop optimization approaches leverage embedded sensors to adjust prosthetic control strategies dynamically [59,60]. Such systems rely on real-time feedback, distributed computing, and edge intelligence to co-adapt human and machine behavior. These adaptive systems are essential for achieving seamless integration between user intent and device behavior, a critical requirement for long-term usability. Among all categories discussed, brain–machine interfaces (BMIs) represent the most clinically mature technology. Wireless cortical implants have entered early-stage human trials (TRL 7+), particularly in paralysis and limb loss applications. However, challenges remain in ensuring bidirectional feedback, longevity of signal quality, and user adaptation. Given ongoing commercial efforts (e.g., Neuralink, Blackrock Neurotech), BMI systems are highly likely to achieve clinical deployment in selected use cases within the next 3–5 years, especially where risk–benefit profiles favor invasive interventions.

**Figure 4:**
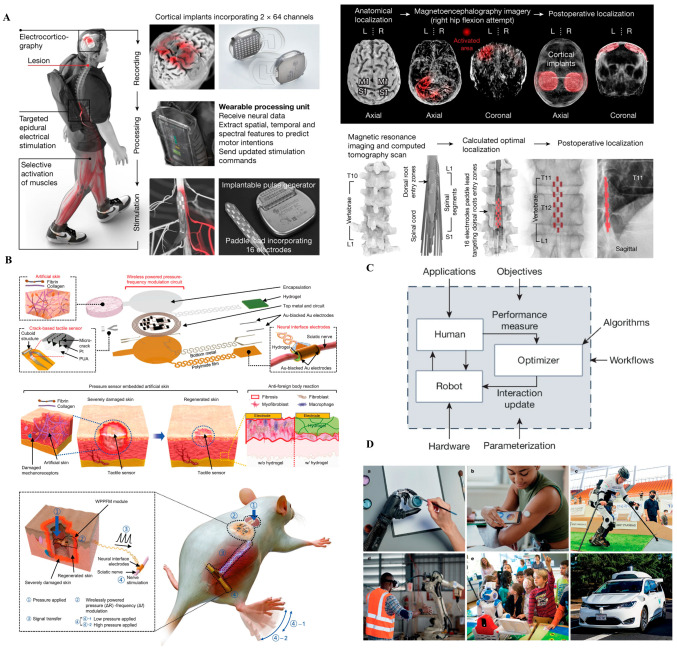
Translational brain–machine interface (BMI) systems. (**A**) Wireless brain–spine interface enabling volitional locomotion after spinal cord injury by decoding cortical signals and delivering targeted spinal stimulation. Reprinted with permission [24]. (**B**) Human-in-the-loop optimization framework for intelligent prosthetic adaptation through real-time feedback and user-involved control tuning. Reprinted with permission from Ref. [19]. Copyright © 2024, Springer Nature. (**C**) Chronically implantable thin-film electrodes delivering somatosensory feedback to the cortex, enabling tactile perception in prosthetic hand users. Reprinted with permission [9]. (**D**) Real-world applications of human–machine systems across rehabilitation, industrial collaboration, assistive robotics, and autonomous mobility, illustrating the transition from laboratory prototypes to clinically and socially integrated platforms.

Closing the loop between decoding and stimulation is essential for restoring meaningful motor and sensory functions, particularly in patients with spinal cord injury or limb loss. Figure 4 presents clinical-stage examples of such bidirectional and adaptive systems, which are further examined below. Figure 4 presents cutting-edge examples of translational brain–machine interface (BMI) technologies that bridge neural decoding, closed-loop feedback, and adaptive control in human subjects. These systems represent a transition from proof-of-concept platforms toward real-world clinical deployment, with a focus on restoring lost function through intelligent and personalized neural interfacing.

Figure 4A depicts a wireless brain–spine interface developed for patients with spinal cord injury (SCI), enabling volitional locomotion by decoding cortical activity and delivering stimulation to spinal circuits in real time. The system integrates intracortical recording arrays, wireless communication, and epidural spinal stimulators. Critically, it uses AI algorithms to map intention to movement patterns. This closed-loop architecture restores meaningful walking ability after complete SCI and sets a benchmark for future neural repair paradigms. Nonetheless, system complexity, calibration burden, and dependence on cortical implants remain non-trivial clinical hurdles [12,25].

Figure 4B illustrates a “human-in-the-loop” optimization platform, where prosthetic adaptation is guided by real-time human feedback and machine learning. Instead of relying on static control strategies, the system learns from user behavior and preference over time, co-adapting to individual needs. This represents a shift from decoder-centered design to bidirectional, user-centered control logic. While highly promising, such platforms require extensive training data, robust adaptation algorithms, and mechanisms to prevent destabilization during use, especially under real-world variability [19].

Figure 4C shows a chronically implantable thin-film electrode array delivering somatosensory feedback directly to the brain, enabling prosthetic users to perceive tactile inputs. This capability closes the loop between action and perception, a fundamental requirement for intuitive prosthesis control. Stimulation of specific cortical sites evokes localized sensations that the user can interpret as pressure or texture. This technology marks a milestone in bidirectional neural prosthetics but still faces limitations in spatial resolution [61,62], perceptual consistency, and long-term tissue response [9].

Figure 4D showcases real-world applications of human–machine interfaces across diverse domains, including rehabilitation, assistive robotics, industrial collaboration, and autonomous mobility. These examples underscore the translational potential of BMI and prosthetic systems beyond laboratory settings. By embedding intelligent control and adaptive feedback, such systems enable personalized assistance, enhance user agency, and support scalable deployment in clinical and non-clinical environments. This progression from technical prototype to human-centered application highlights the importance of usability, robustness, and contextual adaptability in the next generation of neurotechnologies.

Taken together, the systems in Figure 4 exemplify the ongoing evolution from unidirectional decoding architectures to fully bidirectional, co-adaptive human–machine interfaces. This progression reflects increasing technology readiness levels (TRLs 6–8), with several platforms already entering early clinical evaluation. Collectively, these developments mark a critical transition: brain–machine interfaces are no longer confined to experimental settings, but are rapidly becoming clinically viable tools capable of restoring autonomy, enhancing quality of life, and redefining rehabilitation paradigms. Current wireless BMI prototypes transmit up to 20 Mbps at <15 mW total power while achieving noise floors below 10 µV, illustrating a balance between data throughput, energy efficiency, and signal fidelity. However, long-term drift—typically >15% signal loss over six months—remains a persistent limitation (see Supplementary Table S1). Brain–machine interfaces demonstrate advanced fidelity (F) and energy (E) optimization through efficient decoding and wireless powering. However, bio-integration (B) remains incomplete, as cortical arrays still trigger immune encapsulation. Persistent drift (D) in long-term recordings and user adaptation highlights the need for adaptive, closed-loop recalibration.

## 6. Discussion and Outlook

Supplementary Table S2 compiles—under a harmonized reporting standard—the key quantitative metrics and measurement conditions: channel density/pitch, bandwidth, input-referred noise or LOD (with electrolyte, temperature, and electrode area specified), per-channel power at duty cycle, apparent modulus, evidence for chronic lifetime (species, *n*, duration), indicative TRL, and link type. Values are presented as ranges with reference numbers; where full harmonization is not feasible, entries are binned as Low/Med/High with footnoted notes.

Despite rapid progress, critical barriers to clinical translation persist. These include:

- Chronic immune responses and signal degradation;
- Mechanical fatigue from long-term implantation;
- Limitations in material stability, biocompatibility, and packaging durability.

Beyond technical performance, regulatory approval, manufacturing scalability, and standardization of interfaces and data formats remain essential for widespread adoption.

Future research is trending toward intelligent, responsive, and biointegrated systems, with key directions including:

- Self-powered sensors with energy harvesting from body heat or motion;
- Biodegradable closed-loop systems for temporary interventions;
- Soft robotics integration for sensory restoration and neuromorphic computation;
- AI-enhanced decoding algorithms for high-fidelity neural signal interpretation;
- In situ diagnostics using multiplexed chemical sensors for early disease detection;
- Ethical considerations, including privacy, autonomy, and equitable access [63,64,65], must also be integrated into the development and deployment of brain-interfacing implants.

A key message from this review is that the hardest problem ahead is maintaining stable and accurate sensing over time inside living systems. Biological tissues, fluids, and interfaces keep changing, and even small drifts can reduce data reliability. Rather than treating this as an unavoidable problem, future biosensors could turn it into an advantage—by learning from biological feedback and adjusting their response automatically. This adaptive concept may become the next step toward truly long-lasting, intelligent implants.

**Figure 5:**
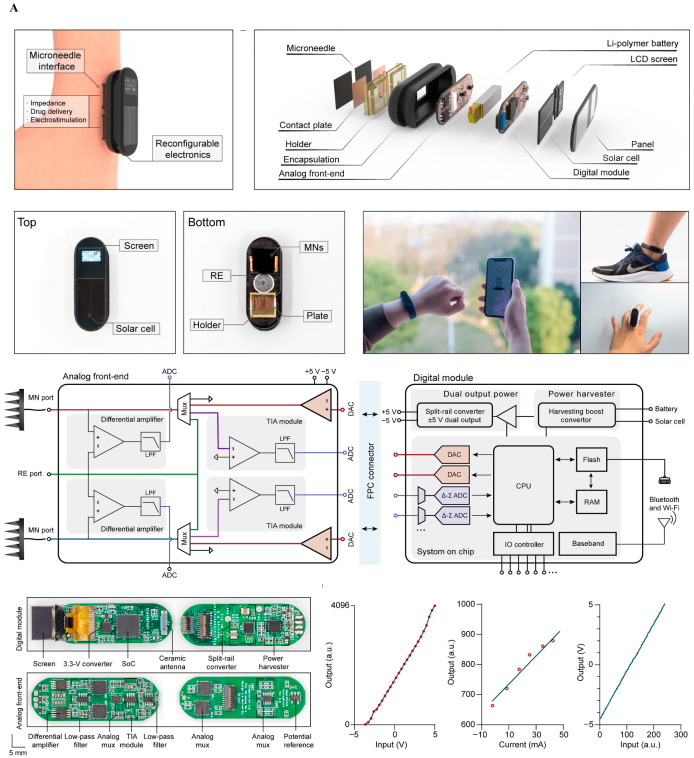

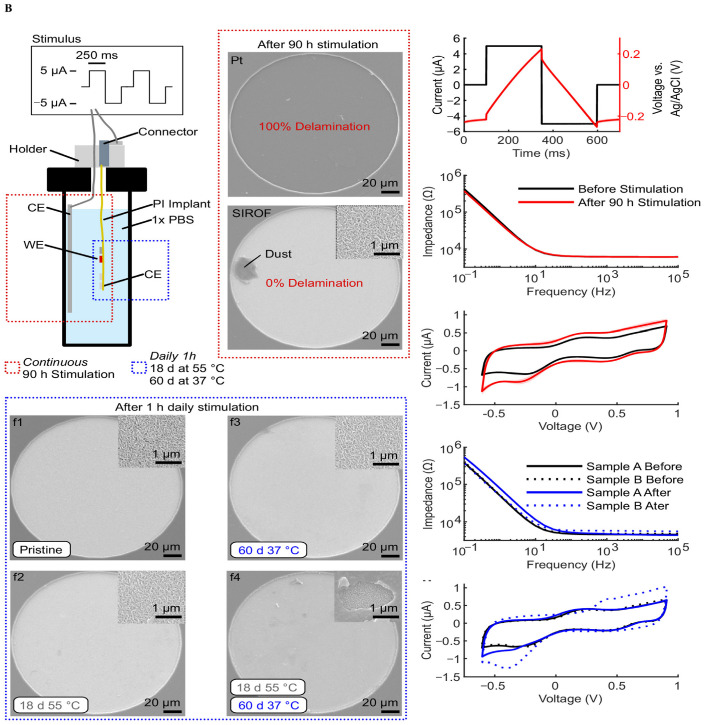

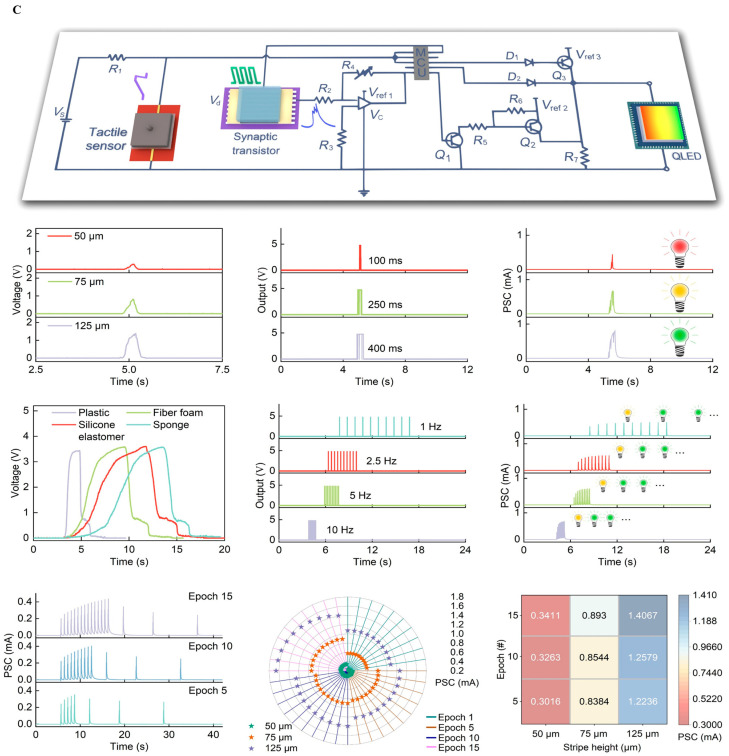
System-level integration of implantable neural interfaces. (**A**) Spider silk–inspired polymer films bridging soft biological tissue and microelectronic interfaces, offering improved mechanical match and biocompatibility. Reprinted with permission from Ref. [21]. Copyright © 2023, Springer Nature (**B**) Wireless water-permeation sensors for real-time encapsulation failure detection, ensuring chronic stability under physiological conditions. Reprinted with permission [8]. (**C**) Hybrid oxide–polymer neuromorphic circuits enabling on-node feature extraction and energy-efficient decision-making in extreme environments. Reprinted with permission [20]. Reprinted with permission from Ref. [26].

Across technologies, reported power densities span 0.1–10 mW cm^−2^ and noise levels range from 5 to 100 µV rms (Supplementary Tables S1 and S2), emphasizing the need to jointly optimize energy efficiency, signal fidelity, and drift resilience within the unified B-FED framework. Figure 5 and Table S1 summarize how emerging implants align with the B-FED framework, linking bio-integration, fidelity, energy, and drift as measurable translational benchmarks. Sustaining high signal fidelity with minimal energy cost—while ensuring stability through integrated drift control—remains the central challenge for next-generation neural interfaces.

These advances require an interdisciplinary ecosystem spanning materials science, microfabrication, neural engineering, and clinical neuroscience to fully realize the therapeutic potential of implantable neurotechnologies (Table S3—Current Challenges and Potential Solutions).

Selective early clinical adoption is most likely in applications where safety envelopes and clinical workflows are already relatively mature. Over the next 3–5 years, progress is anticipated in the following areas: [66,67,68,69,70].

- Wireless spinal or peripheral nerve interfaces with AI-assisted decoding for motor recovery (TRL 7–8);
- Flexible CMOS probes for high-density ECoG mapping (TRL 6–7);
- Human-in-the-loop BMIs enabling real-time adaptive control (TRL 7+).

These advances hinge on demonstrated encapsulation durability, SAR/thermal compliance at true duty cycles, and manufacturability.

By contrast, bioresorbable implants and neuromorphic sensing elements remain early-stage (TRL ≤ 4) and demand further validation—particularly in durability, calibration stability, and verification frameworks—before clinical deployment.

As implantable neural technologies evolve, the focus shifts from component-level innovation to system-level integration, where sensing, stimulation, local computation, and secure wireless links must co-exist under constraints of the following:

- Thermal dissipation/SAR exposure;
- Link bandwidth and latency;
- Drift-induced degradation and calibration costs.

Figure 5 illustrates three archetypal strategies that address these constraints while improving functionality and reducing complexity—each aligned to the B-FED (Bio-integration, Fidelity, Energy, Drift) framework and its five translational gates.

Figure 5A Spider-silk-inspired polymer films bridging soft tissue and electronics—value: improved mechanical match and sterilization compatibility; constraint: barrier reliability under cyclic strain and moisture; report: apparent modulus/thickness and moisture-ingress monitoring thresholds [3,21].

Figure 5B Brain–body–gut interface for concurrent sensing and actuation—value: multi-organ, multimodal closed loop; constraints: cross-domain material/sterilization compatibility and regulatory path; report: link bandwidth/latency at true duty cycles, calibration cadence for chemochannels, and safety envelope [8,16,21].

Figure 5C Moiré-synaptic/neuromorphic array for on-node feature extraction—value: compresses data and heat by moving compute to the sensor; constraints: device variability and verification/validation; report: energy per effective decision, end-to-end Sense → Compute → Act latency, and accuracy/variability at physiological temperature [15,20,26].

Figure 5 illustrates representative implementations of implantable neural biosensors that integrate signal transduction, embedded signal conditioning, and wireless communication. These platforms reflect a shift from isolated measurement units toward bio-integrated, multimodal, and drift-adaptive sensing systems. While some extend to peripheral and visceral targets, their analytical performance—including limit of detection (LOD), response time, drift rate, and stability in physiological environments—remains the primary constraint on translational deployment.

These challenges map directly to the B-FED axes. Bio-integration is enhanced by flexible CMOS and IGT/OECT designs, yet chronic degradation often arises from biofouling, moisture ingress, and micromotion-induced failure rather than intrinsic device limits. Front-end fidelity, particularly in multimodal systems co-recording neurochemical and electrical activity, depends on stable selectivity, low LOD, and minimal drift. Energy and link budgets, constrained by realistic duty cycles, impose thermal and SAR limits; on-node signal conditioning (ratiometric referencing, baseline subtraction, adaptive sampling) can mitigate these, provided analytical accuracy is preserved. Finally, data efficiency is not just algorithmic but determines the feasibility of high-density sensing over chronic time scales.

Figure 5, therefore, underscores the convergence of transduction physics, packaging chemistry, and embedded analytics in the service of biosensing performance and long-term in vivo stability.

To enable condition-explicit and reproducible comparisons, we report the following: (i) LOD and input-referred noise with electrolyte, temperature, and electrode area specified; (ii) response time and drift rate under physiological perturbations (e.g., biofouling, thermal cycling); (iii) chronic survival windows (species, *n*, duration); and (iv) per-channel energy at stated duty cycles and SAR compliance. For multimodal systems, a minimal in vivo calibration set is proposed: LOD drift over time, calibration cadence to maintain accuracy, and post-calibration residuals. These criteria support the benchmark structure in Table S2.

## 7. Materials and Interfaces for Chronic Neural Biosensing

The success of next-generation implantable neural sensors hinges on the thoughtful integration of materials that are biocompatible, mechanically compliant, and compatible with standard microfabrication processes. A central focus lies in identifying materials that match the mechanical properties of neural tissues, minimizing immune response and maximizing recording stability over extended periods.

Among soft polymer substrates, polyimide (PI) and parylene C are widely used due to their excellent chemical stability, thermal resistance, and compatibility with photolithographic patterning. These materials form the structural backbone of many flexible devices, offering thicknesses in the micrometer range that match the modulus of cortical tissue. Meanwhile, liquid crystal polymers (LCPs) and silk fibroin-based materials have emerged for their tunable mechanical properties and biodegradability, respectively, making them attractive for temporary implants.

Encapsulation strategies are critical to protect active circuitry from biofluid ingress while maintaining device flexibility. Advanced thin-film encapsulations, such as alternating layers of Al2O3 and parylene, or SiO_2_/SiNx hybrid structures, offer high barrier properties with minimal thickness. However, evaluating the long-term integrity of these barriers remains a challenge. Innovations such as magnesium-based corrosion sensors embedded beneath encapsulation layers provide real-time, wireless feedback on moisture permeation, offering a route for predictive failure analysis [26,27,28,29,30].

On the conductive layer side, gold (Au), platinum (Pt), and iridium oxide (IrOx) remain standard for neural interfaces, offering low impedance and stable electrochemical characteristics. Emerging materials such as PEDOT:PSS, graphene, and MXenes introduce enhanced charge transfer, transparency, and even mechanical flexibility, positioning them as key candidates for next-generation neural transducers.

Integration of these diverse materials requires advanced fabrication techniques. Microfabrication processes include photolithography, sputter deposition, thermal evaporation, plasma-enhanced chemical vapor deposition (PECVD), and soft lithography. For heterogeneous integration, transfer printing, laser lift-off, and inkjet printing methods allow precise placement of active materials onto flexible or stretchable substrates, enabling complex multilayer devices that retain conformability and performance. We have summarized some representative materials in Table 2.

Additionally, innovations in additive manufacturing, such as 3D printing of bioelectronic inks, are enabling customized device geometries that conform to individual anatomical features. This personalization will likely play an increasing role in patient-specific neural interfaces, where the choice of transduction mechanism—electrical, electrochemical, or ion-gated—must be co-optimized with material properties to ensure signal specificity, stability, and compatibility with chronic implantation [27,28,29,30].

## 8. Toward Clinical Translation and Regulatory Pathways

Despite the impressive progress in lab-scale demonstrations, transitioning implantable neural sensors into clinical use demands careful consideration of biocompatibility, reliability, and manufacturability under regulatory guidelines. The U.S. Food and Drug Administration (FDA), European Medicines Agency (EMA), and other regulatory bodies require stringent safety and efficacy data, particularly for chronic implants interacting with the central nervous system.

Biocompatibility testing includes cytotoxicity, hemocompatibility, immunogenicity, and long-term implantation studies in animal models. Many soft materials and hydrogels exhibit favorable short-term profiles, but their performance in long-term human applications remains an open question. Similarly, wireless power transfer and data communication methods must meet electromagnetic safety limits and cybersecurity standards.

Clinical translation also depends on scalability and reproducibility. Cleanroom-based microfabrication is not always compatible with large-scale manufacturing. Partnerships between academic researchers and industry are essential to bridge this gap, facilitating technology transfer and early-phase human trials. Several startup companies and research hospitals are already piloting flexible neural interface technologies in patients with spinal cord injuries, Parkinson’s disease, and amputations.

Furthermore, interdisciplinary training programs are needed to equip researchers with expertise spanning neuroscience, materials science, electrical engineering, and regulatory science. A robust translational pipeline, supported by public-private partnerships and standardized protocols, will accelerate the delivery of neurotechnologies from the lab bench to the clinic.

## 9. Conclusions

Implantable neural sensor technologies are redefining how we interface with the brain, merging advances in microelectronics, materials science, and systems neuroscience. From ultra-soft multimodal probes and neuromorphic sensors to wireless bioresorbable stimulators, the spectrum of possibilities continues to grow. To fully harness these innovations, future research must embrace a system-level view—focusing not only on isolated devices but on their integration into broader therapeutic and diagnostic ecosystems. This includes closed-loop feedback, AI-enhanced decoding, and compatibility with digital health platforms.

As these technologies move closer to the clinic, they offer a profound opportunity to reshape care for millions of people affected by neurological conditions—through earlier diagnosis, adaptive therapies, and seamless restoration of function. Realizing the promise of implantable neurotechnologies will require not only continued scientific innovation but also cross-sector collaboration spanning engineering, ethics, and medicine.

## Figures and Tables

**Table 1:** Key Technologies and Representative Innovations.

Technology Type	Representative Devices or Methods	Core Advantages
Flexible Neural Interfaces	CMOS flexible probes, cIGT transistors	Soft, conformal, high-density recording
Wireless & Bioresorbable Systems	Backscatter sensors, transient neurostimulators	Minimized surgical burden, auto-degradation
Multimodal & Neurochemical Sensing	Carbon-coated microelectrodes, MoS_2_ neuromorphic memory	Dual-mode recording: chemical + electrical
Brain–Machine Interfaces (BMIs)	ECoG decoding + spinal stimulation loop	Restores motor function, intention-driven control
Neuromorphic & Edge Computing	Synapse-inspired sensing units	Learning-capable, on-device intelligence

**Table 2:** Key Materials and Their Functional Roles.

Material Type	Primary Function	Application Features
Polyimide (PI) Parylene C	Flexible substrate matching brain tissue	Micron-scale flexibility, microfabrication compatible
Au/Pt/IrOx	Conductive layer for stable neural signals	Low impedance, widely used in interfaces
PEDOT:PSS/MXene/Graphene	Enhanced sensitivity and mechanical flexibility	Conductive and optically transparent
Al_2_O_3_/SiO_2_ encapsulation films	Protect circuitry and block biofluid	Barrier quality still under long-term evaluation
Magnesium/Silk/Liquid Crystal Polymers	Enable biodegradability or tunable mechanics	Ideal for short-term implants or post-op therapy

## Supplementary Materials

The following supporting information can be downloaded at: https://www.mdpi.com/article/10.3390/bios15110762/s1, Table S1: Translational Framework for Implantable Neural Sensors (B-FED rubric and quantitative benchmarks; Table S2: Cross-technology quantitative summary. Representative ranges compiled from recent reports; values are given with conditions to avoid cross-study misinterpretation. See footnotes for bandwidth, electrolyte/temperature, electrode area, and duty-cycle assumptions; Table S3: Current Challenges and Potential Solutions. References [71,72] are cited in Supplementary Materials.
