# Supplementary material for: Emerging Implantable Sensor Technologies at the Intersection of Engineering and Brain Science
Source: Biosensors (Basel). 2025 Nov 17;15(11):762. doi: 10.3390/bios15110762 (PMC12650648; doi:10.3390/bios15110762)
Supplement: Supplementary file 1 [file biosensors-15-00762-s001.zip › biosensors-3915413-supplementary.pdf]

Table S1. Translational Framework for Implantable Neural Sensors (B-FED rubric and quantitative benchmarks)

Technology type	Exemplars (non-exhaustive)	Decision-relevant strengths (B-FED)	Typical constraints	Indicative use cases
Flexible neural interfaces	CMOS-integrated flexible probes; cIGT/IGT (OECT) arrays	Soft, conformal coverage; high-density front-end gain; reduced wiring/thermal load (**B, F, E**)	Packaging/yield and long-term encapsulation; drift and calibration for IGT/OECT; heat budget under dense sampling	Chronic ECoG/SEEG; mesoscale microcircuit mapping; closed-loop sensing
Wireless & bioresorbable systems	Passive backscatter sensors; transient neurostimulators	Minimal surgical burden; zero-removal; ultra-low power links (**E, B**)	Bandwidth and pose sensitivity (backscatter); finite lifetime/energy; SAR margins	Acute monitoring; peri-operative therapy; temporary neurostimulation
Multimodal & neurochemical sensing	Carbon-fiber/enzyme-functionalized microelectrodes; MoS_2/memristive auxiliaries	Chemical + electrical co-recording; event-triggered sensing; on-node filtering (**F, D**)	Biofouling and selectivity; LOD drift; calibration cadence	Seizure/DA/5-HT dynamics; drug response profiling; closed-loop chemoneuromodulation
Brain–machine interfaces (BMI)	ECoG/MEA decoding with spinal or cortical stimulation	Intention-driven control; pathway restoration (**F, E**)	End-to-end latency/energy budget; robustness and personalization; safety	Motor restoration; communication interfaces; adaptive DBS
Neuromorphic & edge computing	Synapse-inspired sensing units; on-node compression/classification	Data efficiency; bandwidth/heat reduction; drift adaptation (**D, E**)	Algorithm transparency and OTA updates; verification/validation	Event detection; artifact suppression; adaptive sampling

Supplementary Table S1 provides a comparative snapshot of representative implantable neural sensor platforms, organized by technology type, translational strengths, key engineering constraints, and typical use cases. To support practical decision-making, we applied the B-FED rubric—highlighting aspects of **Bio-integration (B), Front-end fidelity (F), Energy and wireless links (E), and Data efficiency (D)**—to indicate platform-specific advantages. The table also cross-references critical translational considerations, including encapsulation longevity, SAR/thermal limits, manufacturability, OTA security, and calibration burden. Together, this overview offers a structured lens for evaluating design trade-offs and aligning engineering choices with clinical application goals.

Table S2. Cross-technology quantitative summary. Representative ranges compiled from recent reports; values are given with conditions to avoid cross-study misinterpretation. See footnotes for bandwidth, electrolyte/temperature, electrode area, and duty-cycle assumptions.

**Platform**	**Modality**	**Spatial resolution (channels ·mm⁻² / pitch)**	**Bandwidth (Hz/kHz)**	**Input-referred noise / LOD (conditions)**	**Power per channel (µW–mW)**	**Apparent modulus (kPa–GPa)**	**Reported lifetime (model)**	**Notes**
Flexible CMOS probes	ECoG/LFP/spikes	**High** (pitch ≈ **50–300 µm** for micro-ECoG; **0.5–5 mm** for clinical grids)	**0.1–7 kHz** (e-phys front-ends)	**~2–10 µV_rms** (0.5–7.5 kHz band); spike front-ends lower with narrowband	**~1–15 µW** (AFE typical)	**~1–5 GPa** (PI/parylene thin films; effective compliance ↑ with thickness ↓)	**Weeks–months** (rodent/NHP reports)	High density; mature packaging & yield
IGT/OECT arrays	E-phys / in-sensor gain	**High** (pitch ≈ **50–200 µm** on flexible substrates)	**≤1–2 kHz** (device transit limits)	E-phys: **~1–20 µV_rms**; Chem LOD: **nM–µM** (PBS, 37 °C, stated area)	**~0.1–5 µW** (bias + readout)	**kPa–MPa** (PEDOT:PSS/Hydrogels/soft polymers)	**Weeks–months** (bench/acute–subchronic in vivo; drift calibration needs explanation)	Good bio-integration; drift/calibration needed
Multimodal chem-elec stacks	NTs + electrophysiology	**Medium–High** (pitch **100–500 µm**)	E-phys **0.1–7 kHz**; Chem **0–10 Hz**	Chem LOD: **sub-µM–µM** (Mark pH/temperature/electrode area)	**~1–20 µW** (Contains chemical channel front end/multiplexing)	**10 kPa–GPa** (Stack material, depending on package)	**Weeks–months** (Chemical channels require regular calibration)	Added calibration burden; fouling sensitivity
Wireless/bioresorbable systems	Pressure/impedance/chem	**Low–Medium** (Mostly sparse channels)	**10–1000 Hz** (Readback/backscatter bandwidth is limited)	—	**nW–µW** (Passive/Backscatter) ; **mW** (Active Transmitter)	**~1–5 GPa** (PLGA/silk protein/thin metal; thin layer bending compliance)	**Days–weeks** (By Material/Environment)	Low surgical burden; lifetime is finite
Active stimulators (with sense)	Stim + sense	—	Acquisition **0.1–5 kHz**; stimulus pulse independent	—	Acquisition **~10–100 µW/ch**; Stimulation mW pulse duty limited	—	**Years** (Clinical-grade systems)	Throughput vs. thermal/SAR headroom trade-off

Table S3. Current Challenges and Potential Solutions.

Challenge	Why it blocks translation (failure mode)	What to report (condition-explicit)	Example mitigations
Biocompatibility & immune response	Inflammation/gliosis → signal loss and instability	Histology and impedance over time; apparent modulus & thickness; micromotion context	Soft substrates; compliant interconnects; anti-fouling/anti-inflammatory coatings; in vivo conditioning
Long-term stability	Encapsulation degradation; interconnect fatigue/corrosion	Moisture-ingress monitoring method & threshold; failure distribution (Weibull); chronic survival (species, n, duration)	Hermetic/ultra-barrier stacks; corrosion monitors; self-healing encapsulants; stress-relief geometries
Energy & wireless link	Thermal/SAR exceedance; insufficient bandwidth/latency	Power per channel @ duty cycle; boundary temperature rise; SAR margin; link BER/throughput	Passive/backscatter where viable; adaptive duty-cycling; on-node compression/classification
Regulatory & clinical translation	Unclear test methods; lack of standards for soft systems	Bench/animal acceptance criteria tied to hazards; traceable TRL rationale	Early regulatory engagement; method standards via consortia; risk-based test plans
Scalability & standardization	Cost/variability; limited reproducibility	Process Cpk/yield; batch-to-batch variance; sterilization compatibility	Transfer printing/3D patterning; design-for-manufacture; supplier qualification
Cybersecurity & OTA	Integrity/availability risks in closed loop	Threat model; authentication/attestation; secure update cadence	Signed firmware; encrypted links; rollback & safe-mode protocols

Supplementary References

71. Wang, X.; Yu, H.; Kold, S.; Rahbek, O.; Bai, S. Wearable sensors for activity monitoring and motion control: A review. *Biomim. Intell. Robot.* **2023**, *3*, 100089 .
72. Shu, Z.; Liu,P.; Cheng, Y.; Liu, J.; Feng, Y.; Zhu, Z.; Yu,Y.; Han, J.; Wu ,J.; Yu, N. A small-sample time-series signal augmentation and analysis method for quantitative assessment of bradykinesia in Parkinson's disease. *Intell. Robot***. 2024**, *4*, 74–86.
